# Factors associated with utilization of long-acting and permanent contraceptive methods among women who have decided not to have more children in Gondar city

**DOI:** 10.1186/s12905-017-0432-9

**Published:** 2017-09-06

**Authors:** Chernet Baye Zenebe, Mulat Adefris, Melaku Kindie Yenit, Yalemzewod Assefa Gelaw

**Affiliations:** 1 Department of Gynecology and obstetrics, University of Gondar, Gondar, Ethiopia; 2 Department of Epidemiology and Biostatistics, University of Gondar, Gondar, Ethiopia

**Keywords:** Utilization of LAPCM, Factors, Completed family size, Gondar city, Northwest Ethiopia

## Abstract

**Background:**

Despite the fact that long acting family planning methods reduce population growth and improve maternal health, their utilization remains poor. Therefore, this study assessed the prevalence of long acting and permanent family planning method utilization and associated factors among women in reproductive age groups who have decided not to have more children in Gondar city, northwest Ethiopia.

**Method:**

An institution based cross-sectional study was conducted from August to October, 2015. Three hundred seventeen women who have decided not to have more children were selected consecutively into the study. A structured and pretested questionnaire was used to collect data. Both bivariate and multi-variable logistic regressions analyses were used to identify factors associated with utilization of long acting and permanent family planning methods. The multi-variable logistic regression analysis was used to investigate factors associated with the utilization of long acting and permanent family planning methods. The Adjusted Odds Ratio (AOR) with the corresponding 95% Confidence Interval (CI) was used to show the strength of associations, and variables with a *P*-value of <0.05 were considered statistically significant.

**Results:**

In this study, the overall prevalence of long acting and permanent contraceptive (LAPCM) method utilization was 34.7% (95% CI: 29.5-39.9). According to the multi-variable logistic regression analysis, utilization of long acting and permanent contraceptive methods was significantly associated with women who had secondary school, (AOR: 2279, 95% CI: 1.17, 4.44), college, and above education (AOR: 2.91, 95% CI: 1.36, 6.24), history of previous utilization (AOR: 3.02, 95% CI: 1.69, 5.38), and information about LAPCM (AOR: 8.85, 95% CI: 2.04, 38.41).

**Conclusion:**

In this study the prevalence of long acting and permanent family planning method utilization among women who have decided not to have more children was high compared with previous studies conducted elsewhere. Advanced educational status, previous utilization of LAPCM, and information on LAPCM were significantly associated with the utilization of LAPCM. As a result, strengthening behavioral change communication channels to make information accessible is highly recommended.

## Background

Accessibility of reproductive health services, including family planning methods is essential strategy to improve maternal health [1–3]. Of these, modern contraceptive methods not only enhance health and well-being but also reduce infant and maternal morbidity and mortality [4]. In Sub-Saharan African countries, where maternal mortality and morbidity is high and pregnancies are seldom planned, most of the women have a higher parity and greater risk of death, following pregnancy and birth [5, 6]. As a result, increasing the utilization of long acting and permanent family planning methods becomes a strategy for reducing maternal mortality. Surprisingly, the proportions of women who want to limit their family size rather than postponing childbearing are rising globally, and the need for long acting and permanent family planning methods is surpassing the short acting contraceptive methods.

Modern family planning involves discussions among couples and service providers on family health and their desires either to limit or space their family [7, 8]. However, only one-quarter of women of reproductive age in Africa use modern methods of family planning [9, 10], especially short term contraceptive methods, such as pills and injectable [11–13]. Long acting family planning methods such as intrauterine devices (IUD) and implant are referred to as long acting reversible contraceptive methods and prevent pregnancy for at least 3 years are useful for couples wishing to space pregnancies, while long acting and permanent family planning methods such as male and female sterilization, can prevent pregnancy for life and are used by couples who have completed child bearing [13, 14]. Despite the great progress in family planning methods over the last several decades, more women worldwide still want to prevent pregnancy, and the job of family planning remains unfinished [4, 15, 16]. Either services or supplies are not yet available adequately or choices are limited [17]. Fear of social disapproval or partners’ objections, worries of side effects, health concerns [18], lack of knowledge about contraceptive options and their use were some of the formidable barriers in the utilization of long acting contraceptive methods [19].

In Ethiopia, fertility rate has declined from 5.4 to 4.8 children per woman between 2005 and 2011 [20, 21]. According to the 2011 EDHS report, modern contraceptive use which is dominated by short-term methods among married women was reported as 29%. Similarly, the overall prevalence of long acting and permanent contraceptive methods (LAPMs) in Ethiopia was 12.3%. In Amhara Region, where the study was conducted, the annual population growth was 2.9%, while the total fertility rate of 5.4 births per woman was higher than the national figure [21]. However, the promotion and utilization of effective family planning methods in countries with high birth rates and limited resources have a potential for improving maternal and child health, and the proportions of users of long acting and permanent family planning methods, such as female and male sterilization, intrauterine devices, and implant are, no doubt, very low in Ethiopia [13]. Therefore, this study assessed factors associated with the utilization of long acting and permanent family planning methods among women visiting health facilities in Gondar city, northwest Ethiopia after they have decided not to have more children.

## Methods

### Study design and setting

An institution-based cross-sectional study was conducted from August to October, 2015, in Gondar city, northwest Ethiopia. The city has eight health centers and one university hospital which provides services to about 5 million people. Out of the 1260 women who used family planning services in 2014, 818 chose the short acting methods, while 442 utilized the long acting options.

### Sample size and sampling procedure

All women in the reproductive age groups who were attending selected government health facilities after deciding not to have more children participated in this study. The minimum sample size was calculated using the single proportion formula, considering the following assumptions: 12.3% of prevalence of long acting and permanent family planning methods in Ethiopia [21], 95% confidence level, 3.5% margin of error, and 5% of non-response rate. The minimum sample size obtained was 355. All women attending family planning services at Poly Clinic, Family Guidance, and the University of Gondar Hospital were interviewed consecutively.

### Data collection tools and procedure

Data were collected using a structured interviewer administered questionnaire. To maintain consistency, the questionnaire was first translated from English to Amharic (the native language of the study area) and retranslated to English by professional translators and public health experts. Six clinical nurses and two health officers were selected as data collectors and supervisors. Two days’ intensive training was given to both groups regarding the objective of the study, the confidentiality of information, and techniques of conducting interviews. The tool was pretested on 5% of the total sample out of the study area to evaluate the acceptability and applicability of the procedures.

### Operational definitions and study variables

The outcome variable, utilization of the long acting and permanent contraceptive methods, was assessed on the key definition of a contraceptive method, which prevents pregnancy for at least 3 years. Women in the reproductive age groups, especially those who have decided not to have more children, used either the long acting or the permanent contraceptive methods, known as intrauterine devices, implants, female sterilization, and vasectomy.

Women’s’ comprehensive knowledge on long acting and permanent family planning method was assessed using key components of long acting and permanent family planning methods, such as the knowledge of respondents on the type of long acting contraceptive they know, knowledge on for how long the long acting and permanent contraceptive methods (IUCD/implant/vasectomy) can prevent pregnancy, the effect of long acting contraceptive on women’s’ sexual life, and breastfeeding, aware of the return of fertility after removal of long acting reversible contraceptives, and the knowledge on whether it can cause irregular bleeding and cancer. Accordingly, knowledge score of respondents was measured using mean score and it is classified as having “adequate” and “inadequate” knowledge of long acting and permanent family planning methods. Similarly, the attitude of respondents was measured using the perceived side-effect or perception of women on the surgical procedure during insertion and removal, attitude on LAPCM from doing physical activities, the perceive cause of long acting contraceptive methods to cause permanent infertility, perception that it should be used by married women, partner involvement to decide the contraceptive use. A likert scale measure, ranging from “strongly disagree” to “strongly agree” was used, and consequently the mean scores were used to categorize the attitude into “favorable” and “unfavorable” attitude.

### Data processing and analysis

Data were entered into Epi-info version 3.5.3, and exported to Statistical Package for Social Sciences (SPSS) version 20 for further analysis. Descriptive statistics, including frequencies and proportions were used to summarize the variables. A binary logistic regression was used. Both bi-variable and multivariable logistic regression analyses were carried out. Variables with a *p*-value of less than 0.2 in the bi-variable analyses were entered into the multivariable analysis. Both Crude Odds Ratio (COR) and Adjusted Odds Ratio (AOR) with 95% confidence intervals were estimated to show the strengths of associations. Finally, a *p*-value of less than 0.05 in the multivariable logistic regression analysis was used to identify variables significantly associated with long acting and permanent family planning method utilization.

## Results

### Socio-demographic and economic characteristics

A total of 317 women aged between 15 and 49 years and have decided not to have more children were included in the study, with a response rate of 91%. The mean age of the participants was 36 years. Nearly three-quarter (71.3%) of the women were urban dwellers and more than half (59%) had 5 and less children. More than one-third (38.8%) were illiterate. Over half (54.9%) were housewives. The majorities (97.8%) of the respondents were married, and about 71% were Orthodox Christians (Table 1).

**Table 1:** Socio-demographic characteristics of women who have completed their family size in Gondar town, northwest Ethiopia, 2015

Variables	Frequency	Percent
Mothers’ age (year)		
28-33	89	28.1
34-39	162	51.1
40-45	57	18.0
≥ 46	9	2.8
Residence		
Rural	91	28.7
Urban	226	71.3
Monthly income (ETB)		
≤ 500	47	14.8
501-1000	105	33.1
≥ 1001	165	52.1
Number of children		
< 5	187	59.0
≥ 5	130	41.0
Number of abortions		
1	200	63.1
≥ 2	117	36.9
Educational level		
No formal education	123	38.8
Primary	69	21.8
Secondary	78	24.6
College and above	47	14.8
Occupation		
Farming	47	14.8
Housewife	174	54.9
Self-employee	49	15.5
Government employee	47	14.8
Marital status		
Single	1	0.3
Married	310	97.8
Divorced	4	1.3
Separated	2	0.6
Religion		
Orthodox	225	71.0
Muslim	92	29.0

### Information source of LAPM among women of reproductive age groups

The majority (83.6%) of the women in the reproductive age group (15-49 years) who have decided not to have more children heard about long acting and permanent family planning methods. The main sources of information about LAPM were health professionals (82.6%), television programs (47.3%), and the radio (40.1%). Newspapers, books, and leaflets had limited contribution to disseminating information on LAPM. One-third (34.7%) of the women had no adequate knowledge on long acting and permanent family planning method (Table 2).

**Table 2:** Sources of information, knowledge and attitude on long acting and permanent family planning methods for mothers who have completed their family size in Gondar town, northwest Ethiopia, 2015

Variables	Frequency	Percent
Heard about long acting and permanent family planning methods		
Yes	265	83.6
No	52	16.4
Source of information^a^		
Health workers	262	82.6
Television	150	47.3
Radio	127	40.1
Newspaper	12	3.8
Leaflets	10	3.2
Magazine	6	1.9
Knowledge on LAPM		
Adequate	207	65.3
Inadequate	110	34.7
Attitude on LAPM		
Favorable	187	59.0
Unfavorable	130	41.0

^a^Multiple responses are possible

**Table 3:** Reasons of not using long acting and permanent family planning methods among mothers who have completed their family size in Gondar town health facilities, northwest Ethiopia, 2015

Variables	Frequency	Percent
Reason for not using LAPCM currently^a^		
Fear of side effects	174	54.9
Lack of knowledge	110	34.7
Fear of Procedure	68	21.5
Husband disapproval	49	15.5
Using other methods	27	8.5
Religious prohibition	15	4.7
Opposed LAPCM	6	1.9

^a^Multiple responses are possible

**Table 4:** Factors associated with long acting and permanent family planning methods among women who have completed their family size in Gondar town health facilities, northwest Ethiopia, 2015

	LAPCM Utilization		Crude Odds Ratio	Adjusted Odds Ratio
Variables	Yes	No	(95% CI)	(95% CI)
Age				
28-33	51	38	1	1
34-39	36	126	2.73 (2.05, 5.93)	1.54(0.92, 3.57)
≥ 40	24	42	1.95 (1.12, 3.86)	1.74 (0.76, 2.14)
Residence				
Rural	21	70	0.46 (0.26, 0.81)	0.65 (0.35, 1.69)
Urban	89	137	1	1
Educational status				
No formal education	25	98	1	1
Primary	23	46	1.96 (1.01,3.82)	1,66(0.81,3.38)
Secondary	37	41	3.54 (1.89,6.61)	2.28(1.17, 4.44)*
College and above	25	22	4.45(2.16,9.16)	2.91(1.36,6.24)*
Occupation				
Farming	12	35	0.66(0.28,1.62)	0.34(0.13,2.38)
Housewife	52	122	0.83(0.42,1.62)	0.74(0.32, 1.45)
Government employee	30	19	3.06(1.33,7.04)	1.91(0.64,5.33)
Self-employee	16	31	1	1
Monthly income (ETB)				
≤ 500	28	19	4.12(1.89, 8.45)	2.34 (0.53, 6.31)
501-1000	76	29	7.44(2.35, 11.02)	3.87(0.68, 5.91)
≥ 1001	24	141	1	1
No of children				
< 5	79	108	2.34(1.42, 3.84)	1.85 (0.72, 2.68)
≥ 5	31	99	1	1
Information about LAPCM				
Yes	87	157	17.19(4.09,27.18)	8.85(2.04, 18.41)*
No	21	50	1	1
Previous use of LAPCM				
Yes	42	28	3.95(2.27,6.86)	3.02(1.69, 5.37)*
No	68	79	1	1

^*^Variables with *P*-value of ≤0.05

### Prevalence of utilization of long acting and permanent family planning methods

In this study, the level of utilization of long acting and permanent family planning methods was 34.7% (95% CI: 29.5-39.9). Of these, nearly one-third (28.4%) used implants, and the rest (5.7% and 0.6% respectively) used IUD and female sterilization (tuba-ligation). Out of the total 207 (65.3%) respondents who were using short acting family planning methods, half (50.5%) were using the injectable method. The most reported reasons for not using the long acting methods for 54.9% and 34.7% of the women were fear of side effects and lack of knowledge, respectively. The proportion of women who were using the longer and permanent methods was relatively high (51.1%) among the 34-39 years age group (Table 3).

### Factors associated with utilization of long acting and permanent family planning methods

In the bivariable logistic regression analysis, residence, number of children, occupation, educational status, age, monthly income, information about LAPCM, and previous use of LAPCM were factors associated with the utilization of long acting and permanent family planning methods at a *p*-value of less than 0.02. Consequently, these variables were entered into the multi-variable logistic regression analysis, and it was noted that educational status, information about LAPCM, and previous use of LAPCM were factors associated with the utilization of long acting and permanent family planning methods at *p*-value of less than 0.05.

The odds of long acting and permanent family planning methods among women with secondary level education were 2.28 times higher compared with women who had no education (AOR: 2.28; 95% CI: 1.17-4.44). Similarly, the odds were almost three times higher among women with college and above educational status (AOR: 2.9; 95% CI: 1.36-6.24). Moreover, high odds of long acting and permanent family planning method utilization was noted among women who had information about LAPCM compared with women who had no such information (AOR:8.85; 95% CI: 2.04- 38.41). In addition, women who had previous history of using long acting family planning methods were more likely to use long acting and permanent family planning methods than those who had no history of using LAPCM. In this study, the odds of using long acting and permanent family planning methods among women who had previous history of using LAPCM were three times higher than those of women who had no such history (AOR: 3.01; 95% CI: 1.69,5.38) (Table 4).

## Discussion

In Ethiopia although contraceptive utilization growth has grown rapidly, women mainly relied on short-term family planning methods. Accordingly, the Government has taken initiatives to increase access to long acting and permanent methods. Therefore, this study assessed the utilization of LAPCM and associated factors among women attending health facilities in Gondar city after deciding not to have more children.

In this study, the overall utilization of LAPCM among reproductive age women who have completed their family size was 34.7% (95% CI: 29.5-39.9). The finding was higher than the EDHS report [21],and those of studies done at Mekelle (12.3%) [13], Arbaminch (13.1%) [22], Debremarkos (19.5%) [23], Nekemete (20%) [24], Goba (18.1%) [11], and Jinka (7.3%) [25]. The better utilization of permanent family planning methods noted in this study compared to the studies mentioned above may be due to the fact that a large number of our participants were urban dwellers who had more access to information. Besides the recent expansion of the Urban Health Extension Program in the study area might have played its role.

Out of the variables which showed significant associations at the multi-variable logistic regression analysis, high odds of using long acting and permanent family planning methods were seen among women with secondary and college level educational status. This finding suggests that women with higher education are more likely to go for family planning services than women with lower education. This finding was consistent with those of other studies conducted in Mekele, Arbaminich, and Nekemte [13, 22, 24]. This might be due to the fact that better educated women would be more likely to have access to information on modern contraceptive methods, better knowledge about modern contraceptives and are more likely to use the services. In addition, the educational refinement of secondary school and above educated women may influence their service utilization and decision-making power on reproductive health issues, including family planning. Furthermore, health workers reported as the major source of information in this study might communicate the message on long acting contraceptive methods in a more effective manner than the other possible media.

Consistent with previous studies [11, 13, 22, 23, 25, 26], the odds of using long acting and permanent family planning were higher among women who had information on LAPCM. Women who had information about LAPCM were almost nine times more likely to use long acting and permanent contraceptive methods compared with women who had no information (AOR = 8.85, 95% CI: 2.04 38.41). Our finding was consistent with those of studies conducted in Ethiopia, for example, at Jinka, Hosana, and Mekelle. This might be due to the dissemination of information on television and the radio increasing knowledge and awareness on new ideas, social changes, and opportunities which affect individual perceptions and behavior. Community-based studies in Jinka and Mekelle also revealed that higher odds of the utilization of long acting and permanent family planning methods were noted among women who had knowledge of modern contraceptive methods. Moreover, the rising number of trained health professionals in the last three to 4 years might have contributed to this variation among the studies. Similarly, in some other studies conducted elsewhere [24, 26], women who had history of using LAPCM had higher odds of using long acting and permanent family planning methods.

This study has attempted to show the prevalence of long acting and permanent family planning method utilization, particularly among mothers who have decided not to have more children. However, the study is not free from limitations. For example, our sample size, we feel, is inadequate. Besides, we were unable to use the qualitative method of study. On top of that, social desirability bias is the other possible limitation of our study. Our inability to reach conclusions which can be generalized to the wider community can also be the limitation of our work.

## Conclusion

In this study, utilization of long acting and permanent family planning methods was higher compared with reports of previous studies. Among the variables, secondary and above educational level, previous history of use, and information on LAPCM were significantly associated with it. Therefore, strengthening reproductive health information through information, education, and communication is highly recommended.

## Abbreviations

AOR: Adjusted odds ratio
CI: Confidence interval
COR: Crude odds ratio
EDHS: Ethiopia demographic and health survey
IUD: Intrauterine device
LAPCM: Long acting and permanent family planning method
MDG: Millennium development goal
SPSS: Statistical package for social sciences
WHO: World Health Organization
